# Supplementary material for: Characterizing Cell-Free Transcription and Translation Dynamics with Nucleic Acid–Based Assays
Source: ACS Synth Biol. 2025 Dec 24;15(1):243–61. doi: 10.1021/acssynbio.5c00677 (PMC12814534; doi:10.1021/acssynbio.5c00677)
Supplement: Supplementary file 1 [file sb5c00677_si_001.pdf]

Nucleic Acid-Based Assays for Characterizing Cell-Free Transcription and Translation Dynamics

Supplementary File I

Fernanda Piorino[1,2,*], Chad Sundberg[3], Elizabeth A. Strychalski[1], Eugenia Romantseva[1]

[1] National Institute of Standard and Technology; Gaithersburg, MD, 20899, USA.
[2] Johns Hopkins University; Baltimore, MD, 21218, USA.
[3] University of Maryland, Baltimore County; Baltimore, MD, 21250, USA.

* Correspondence: fernanda.piorinomacruzdeoliveira@nist.gov

Table of Contents

I. Nucleic acid templates 2
II. Measurement calibration 3
III. Cell-free extracts and reactions 4
IV. Data processing and analysis 8
V. Additional data 10
VI. References 19

I. Nucleic acid templates

Table S1: Nucleic acid templates used in each experiment.

Figure	Nucleic acid template	Type	Concentration (nmol/L)
2A-C, left	pJL1, pFP34, pFP35	DNA	5
2A-C, center	pFP34, pFP34ΔsfGFP, pFP34ΔRBS	DNA	5
2A-C, right	pFP35, pFP35ΔsfGFP, pFP345ΔRBS	DNA	5
3A-C, left	pJL1, pFP34, pFP35	RNA	300
3A-C, center	pFP34, pFP34ΔsfGFP, pFP34ΔRBS	RNA	300
3A-C, right	pFP35, pFP35ΔsfGFP, pFP345ΔRBS	RNA	300
4B-D, left	pFP34	DNA	5
4B-D, center	pFP34	DNA	5
4B-D, right	pFP35	DNA	5
5B	pFP24	DNA	5
5C	pFP59	DNA	5
5D	pFP60	DNA	5
6A-B, left	pFP34	DNA	5
6A-B, center	pFP34	DNA	5
6A-B, right	pFP35	DNA	5
7A	pFP34	DNA	5
7B	pFP35	DNA	5

Table S2: Primers used to linearize plasmids prior to *in vitro* transcription.

	Primer sequence (5′ to 3′)
Forward primer	TGTCGGGTTTCGCCACCTC
Reverse primer	CAGTTTCATTTGATGCTCGATGAGTTTTTC

II. Measurement calibration

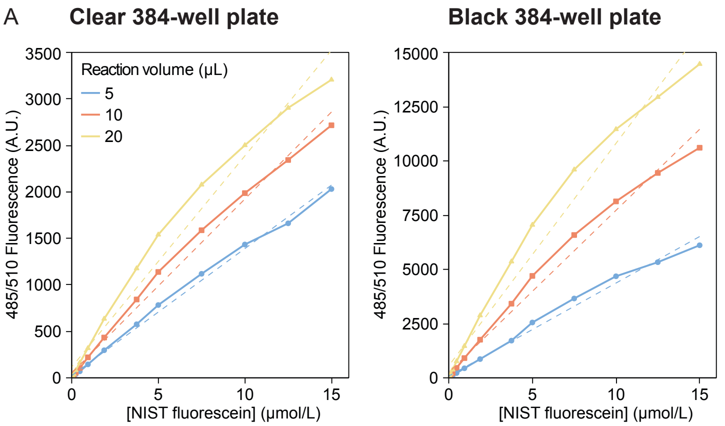

Plate	Reaction volume (µL)	Gain	Slope	Y-intercept
Clear 384-well	5	75	2562.95	9.81
Clear 384-well	10	75	3506.37	7.74
Clear 384-well	20	75	4252.75	8.77
Clear 384-well	5	50	136.53	0.67
Clear 384-well	10	50	187.14	0.14
Clear 384-well	20	50	227.15	0.58
Black 384-well	5	50	428.81	1.29
Black 384-well	10	50	747.14	0.48
Black 384-well	20	50	1025.55	1.67

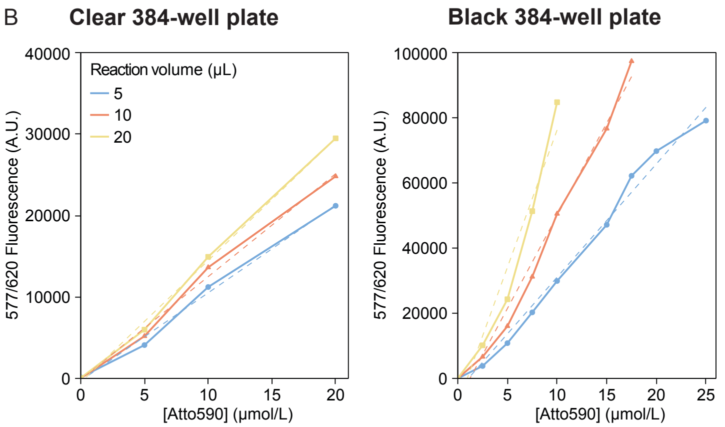

Plate	Reaction volume (µL)	Slope	Y-intercept
Clear 384-well	5	1078.55	133.31
Clear 384-well	10	1263.49	30.56
Clear 384-well	20	1496.3	53.38
Black 384-well	5	3477.99	7.85
Black 384-well	10	5692.36	4.23
Black 384-well	20	8427.9	5.92

Figure S1. Calibration curves for the fluorochromes used in this work. **(A)** NIST-traceable fluorescein standard at gain 50 and **(B)** Atto 590 at gain 100, each at 5, 10 or 20 µL reaction volumes in either a clear 384-well plate (Greiner Bio-One, 784101) or a black 384-well plate (Corning, 3544). The dashed lines were generated via linear regression. The tables indicate the slope and y-intercept values used to convert fluorescence intensity values to Molecules of Equivalent Soluble Fluorochrome (MESF) in µmol/L in all the experiments presented in this study. The slope value was taken directly from the linear regression, and the fluorescence value for a measurement of buffer (sodium borate for NFS or DMSO for Atto 590) lacking the fluorochrome was used as the y-intercept.

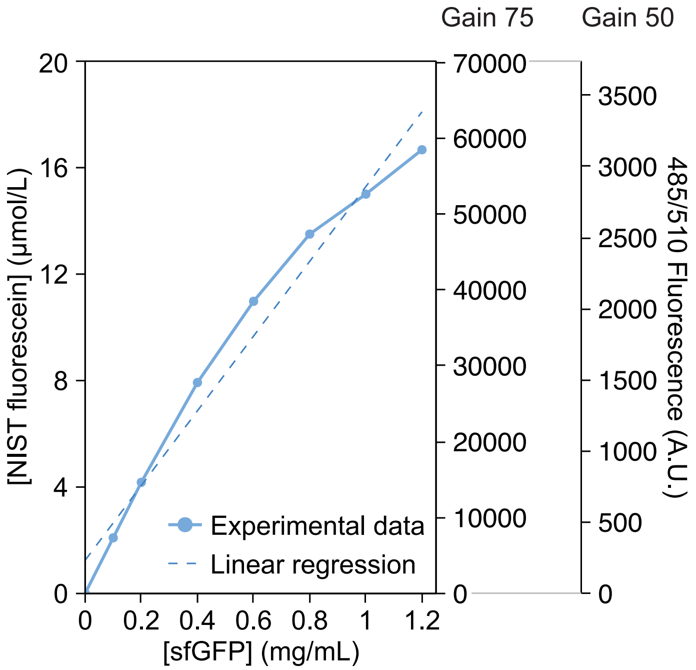

Fluorescence = slope×[sfGFP] + y-intercept

Gain	Slope	Y-intercept
75	49230.73	4386.36
50	2627.23	228.87

Figure S2. Calibration curve for purified sfGFP-His6. Purified protein was added at different concentrations to a 10 µL cell-free reaction in EXT3 in a clear 384-well plate. Fluorescence intensity measurements were taken at gains 75 and 50 as described in the *Methods* section of the manuscript (excitation: 485 nm, emission: 510 nm, bandwidth: 10 nm).

III. Cell-free extracts and reactions

Bacterial genotypes

BL21 (DE3): F– *ompT* *hsdS*$_B$ (rB–, mB–) *gal dcm* (DE3)

BL21 Star (DE3): F–ompT hsdSB (rB–, mB–) gal dcm rne131 (DE3)

Where (DE3) = λ sBamHIo ΔEcoRI-B int::(lacI::PlacUV5::T7 gene1) i21 Δnin5

Extract preparation

The Methods section of the manuscript describes the protocol used to prepare EXT3. To prepare the other extracts characterized in this study, we modified this baseline protocol, as listed in Table 1. Below are more details on the protocol modifications for each extract.

- **EXT10:** Prior to lysis, the cell resuspension was divided into 1 mL aliquots in 1.5 mL microcentrifuge tubes. Tubes were placed in a pre-cooled rack (Qsonica, 514), and samples were lysed simultaneously using a Q700 sonicator (Qsonica) with a 24-tip horn. Each tip has a 3.175 mm diameter, matching the single tip of the Q125 sonicator (Qsonica) used to prepare all other extracts. While Q700 was programmed with the same settings as Q125 (20 kHz frequency, 50 % amplitude, cycles of 10 s on and 10 s off), Q700 did not display the sonication energy input delivered by each tip; instead, it displayed the total energy input, which was lower than the target input per tube or tip (around 260 J) when divided by the number of tubes. Because tubes were placed in a rack during sonication, they could

not be moved up and down, likely resulting in more uneven lysis than for other extracts. In addition, fewer than 24 tubes were used at a time, so tubes filled with water were placed in the empty slots in the rack. We previously noticed that samples on the edges of the rack had different lysis efficiencies than samples in interior slots of the rack; when preparing EXT10, we did not place samples on the edges of the rack to avoid this issue.

- **EXT12:** The extract was prepared from *E. coli* BL21 (DE3) cells lacking the Star (rne131) mutation.
- **EXT14:** After harvest, cells were resuspended and washed in S30A buffer with a pH adjusted to 10 instead of 7.7. 1 mL of this buffer was added per g of cells prior to lysis.
- **EXT15:** Cell cultures were allowed to grow until reaching an optical density of 2.0 at a wavelength of 600 nm (OD600).
- **EXT16:** Cell cultures were allowed to grow until reaching an OD600 of 2.2.
- **EXT17:** After harvest, cells were resuspended and washed in S30A buffer instead of S30 buffer. 1 mL of S30A was added per g of cells prior to lysis.
- **EXT18:** After harvest, cells were resuspended and washed in S30A buffer instead of S30 buffer. 1 mL of S30A was added per g of cells prior to lysis. After cell lysis and centrifugation, the supernatant was isolated and used directly in cell-free reactions without undergoing a runoff reaction.
- **EXT19:** Cell cultures were allowed to grow for 24 h prior to harvest.
- **EXT20:** After cell lysis and centrifugation, the supernatant was isolated and used directly in cell-free reactions without undergoing a runoff reaction.
- **EXT23:** During cell growth, no IPTG was added to induce expression of T7 RNAP.

Reaction formulation

To facilitate reaction assembly and ensure reagent integrity, reagents for cell-free reactions were prepared by grouping them into multiple concentrated solutions. A stock solution of HBC620 was prepared by dissolving HBC620 powder in DMSO.

Table S3. Cell-free reaction formulation.

Reagent	Composition	Stock concentration	Final concentration	Unit
Solution I	Magnesium glutamate	150	10	mmol/L
	Potassium glutamate	2000	133.3	mmol/L
	Ammonium glutamate	150	10	mmol/L
Solution II	ATP	18	1.2	mmol/L
	CTP	12.75	0.85	mmol/L
	GTP	12.75	0.85	mmol/L

	UTP	12.75	0.85 mmol/L
	Folinic acid	0.51	0.034 mg/mL
	tRNA	2.565	0.171 mg/mL
**Solution III**	NAD	3.96	0.33 mmol/L
	Coenzyme A	3.204	0.267 mmol/L
	Sodium oxalate	48	4 mmol/L
	Putrescine	12	1 mmol/L
	Spermidine	18	1.5 mmol/L
	HEPES	600	50 mmol/L
**Amino acids**	20 standard AAs	50	2 mmol/L
**Energy molecule**	PEP	1000	30 mmol/L
**HBC620**	HBC620	1000	5 µmol/L
**Extract**	Extract	-	26.7 % (v/v)

In the experiment described in Figure S13, pyruvate was added to the relevant reactions at a concentration of 30 mmol/L in addition to or in place of PEP.

Reaction preparation
Nucleic acid templates and water were mixed in PCR tubes on ice. A "master mix" of solutions I-III, amino acids, HBC620 dye, and the energy molecule was prepared separately in a microcentrifuge tube, mixed by vortexing, then divided into PCR tubes. Reactions were mixed by vortexing, then 10 µL of each reaction were added to each of 3 wells (constituting technical triplicates) of a 384-well plate stored at room temperature.

Reaction formats
Unless otherwise specified, reactions were run at 10 µL volumes in a clear 384-well plate (see specifications below) incubated at 37 ˚C in a microplate reader without shaking. Plates were sealed with an adhesive film. Plate dimensions and drawings can be found on the manufacturer's website. Two types of plate were used:

- Greiner Bio-one, catalog number 784101: non-binding, clear plate with a clear, flat bottom, and a working volume of 4 µL to 25 µL. Each well can hold up to 28 µL.

- Corning, catalog number 3544: non-binding, black plate with a clear, flat bottom, and a working volume of 5 µL to 40 µL. Each well can hold up to 50 µL.

Table S4: Reagents used to prepare extracts and cell-free reactions.

Reagent	Manufacturer	Catalog number
Tryptone	Fisher Scientific	BP1421-500
Sodium chloride	Millipore Sigma	S3014-500G
Yeast extract	Fisher Scientific	BP1422-500
Potassium phosphate dibasic	Millipore Sigma	60353-250G
Potassium phosphate monobasic	Millipore Sigma	P9791-100G
Isopropyl β-D-1-thiogalactopyranoside (IPTG)	Millipore Sigma	I6758-5G
Trizma® base	Millipore Sigma	T6066-500G
Acetic acid	Millipore Sigma	695092-100ML
Dithiothreitol (DTT)	Thermo Fisher (Invitrogen)	15508013
HBC620	MedChemExpress	HY-133520
Coenzyme A sodium salt hydrate (CoA)	Millipore Sigma	C3144-25MG
β-Nicotinamide adenine dinucleotide (NAD)	Millipore Sigma	N8535-15VL
tRNA from *E. coli* MRE600	Millipore Sigma (Roche)	10109541001[1]
L-Glutamic acid hemimagnesium salt tetrahydrate	Millipore Sigma	49605-250G
L-Glutamic acid potassium salt monohydrate	Millipore Sigma	G1149-500G
L-Glutamic acid monoammonium salt	Toronto Research Chemicals	TRC-G597140
Folinic acid	Millipore Sigma	F7878-100MG
Spermidine	Millipore Sigma	85558-5G
1,4-Diaminobutane (putrescine)	Millipore Sigma	D13208-25G
HEPES	Thermo Fisher	11344041
Potassium oxalate monohydrate (oxalic acid)	Millipore Sigma	P0963-100G
NTP Set	ThermoFisher	R0481
Potassium hydroxide	Fisher Scientific	SP236-500
Phosphoenolpyruvate (PEP)	Millipore Sigma	860077-250MG
Pyruvate	Millipore Sigma	P5280-25G
L-Aspartic Acid	Millipore Sigma	A7219-100G
L-Valine	Millipore Sigma	V0500-25G
L-Tryptophan	Millipore Sigma	T0254-25G
L-Phenylalanine	Millipore Sigma	P2126-100G
L-Isoleucine	Millipore Sigma	I2752-25G

L-Leucine	Millipore Sigma	L8000-25G
L-Cysteine	Millipore Sigma	C7352-25G
L-Methionine	Millipore Sigma	M9625-25G
L-Alanine	Millipore Sigma	A7627-100G
L-Arginine	Millipore Sigma	A8094-25G
L-Asparagine	Millipore Sigma	A0884-25G
Glycine	Millipore Sigma	G7126-100G
L-Glutamine	Millipore Sigma	G3126-250G
L-Histidine	Millipore Sigma	H8000-25G
L-Lysine	Millipore Sigma	L5501-25G
L-Proline	Millipore Sigma	P0380-10G
L-Serine	Millipore Sigma	S4500-100G
L-Threonine	Millipore Sigma	T8625-25G
L-Tyrosine	Millipore Sigma	T3754-100G

[1]tRNA from *E. coli* MRE600, previously manufactured by Roche and widely used for cell-free gene expression, has been discontinued. Sigma Aldrich provided another commercially available tRNA product, but from *E. coli* W (catalog number R1753). We have observed similar sfGFP yields from pJL1 with the two tRNA sources, although stronger cell-free expression is reported with Roche's tRNA product in a reconstituted system prepared in house[1]. At the time this manuscript was written, Sigma Aldrich's tRNA product was no longer available for purchase.

IV. Data processing and analysis

Data processing

We opted to use "signal" to refer to sfGFP and Pepper expression data because the measurable fluorescence intensity, which we later converted to Molecules of Equivalent Soluble Fluorochrome (MESF), encompasses multiple processes. For sfGFP, the increase in fluorescence intensity reflects the transcription, translation, and maturation of sfGFP, including protein folding and formation of the fluorescent chromophore. For the Pepper RNA aptamer, the increase in fluorescence intensity results from transcription, folding, and binding to the HBC620 dye.

We used a Python script to process sfGFP and Pepper-HBC620 fluorescence intensity data and compute metrics for cell-free reactions. Our Python script followed the steps summarized below.

1) Compute the average of three technical replicates and their standard deviation.
2) Convert the average fluorescence intensity and corresponding standard deviation to MESF.
3) For each reaction, subtract the MESF for a reaction containing all reagents except added nucleic acid templates from the average MESF.
4) Compute reaction metrics.

Computation of reaction metrics

The values of all computed reaction metrics are listed in the Reaction Metrics sheet of Supplementary File 2. For sfGFP expression data, we computed the maximum signal, the time to the maximum signal, the maximum rate of signal increase, and time to the maximum rate of signal increase. Once the sfGFP signal plateaued, we did not observe a decrease in signal in any experiments. For Pepper expression data, we included two additional metrics—the maximum rate of signal decrease and the time to reach that rate—to account for the degradation of Pepper RNA.

To calculate reaction metrics, we first generated plots for the derivatives of the sfGFP and Pepper signals. We used the "signal" function from the SciPy library with a Savitzky-Golay filter ("savgol_filter") to generate an array of derivative values. As the function's inputs, we specified a window length of 5, a polynomial order of 1, a derivative order of 1, and a delta value equal to the time interval between measurements. We then computed the metrics below.

- **Maximum signal**: Computed as the maximum average MESF value, and also as the signal measured when two sequential derivative values differed by less than the associated standard deviation.
- **Time to maximum signal**: The time at which the maximum signal was measured.
- **Maximum rate of signal increase**: The maximum derivative value.
- **Time to the maximum rate of signal increase**: The time at which the maximum rate of signal increase was measured.
- **Maximum rate of signal decrease:** The minimum derivative value.
- **Time to the maximum rate of signal increase**: The time at which the maximum rate of signal decrease was measured.

For sfGFP measurements, the maximum signal is often referred to as the "endpoint" signal, due to negligible protein degradation in BL21 extracts and PURE systems. For Pepper measurements, the maximum signal marks the transition from a regime of signal increase to a regime of signal decrease, when RNA degradation exceeds Pepper aptamer transcription, folding, and binding to the HBC620 dye.

Measurements of both maximum signal and rate are needed to provide a complete picture of a reaction and enable accurate comparison of different systems. For example, reactions may have the same maximum rate of sfGFP signal increase, but one may sustain higher rates for longer than the other, resulting in a higher maximum sfGFP signal (Figure 4D, left). While the maximum rates of signal increase and decrease do not capture full reaction dynamics over the course of a reaction, these metrics simplified our analysis and provided additional insight into reaction longevity and tradeoffs between transcription and translation.

As we did for all other reactions, we computed the maximum rate of signal increase for reactions with RNA templates in EXT3, although the computed rate does not reflect an actual increase in signal. This value results from minor noise in the data.

Temperature-related effects on fluorescence measurements
In some experiments, measurements of sfGFP and Pepper signal exhibited a decrease in signal in the first (30 to 40) min of the reaction. Such a decrease is typical of the reaction format used in this work and can likely be attributed to the change in temperature as the reaction vessel equilibrates to 37 ˚C in the plate reader. This feature is more commonly observed for translation, which has a longer lag than transcription. However, we also observed an initial decrease in Pepper signal with most ΔsfGFP and ΔRBS DNA templates, likely because processes leading to an increase in Pepper signal (transcription, folding, and binding to the HBC620 dye) were not sufficiently strong to mask the temperature-related signal decrease (Figure 3A, center and right). This initial signal decrease and the subsequent signal increase interfered with the calculation of reaction metrics, so for certain reactions we excluded these data points from our analysis.

V. Additional data

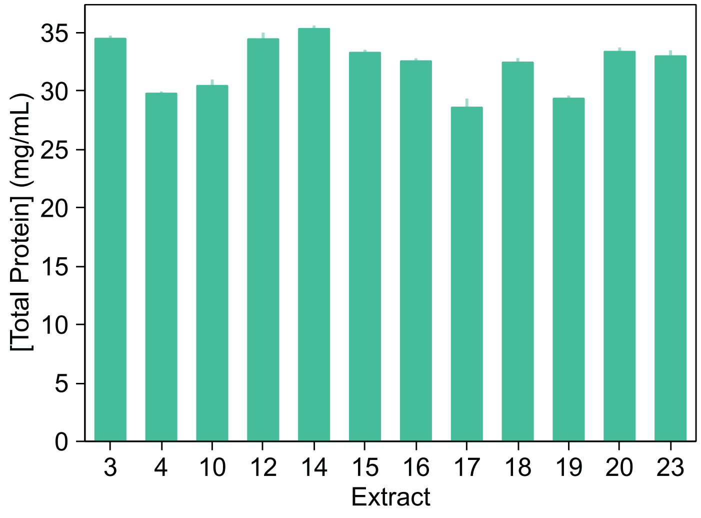

Figure S3. Total endogenous protein content of extracts prepared in house, measured via Bradford assay. Details of the assay are described in the *Methods* section in the main text. The error bars indicate the standard deviation of three technical triplicates.

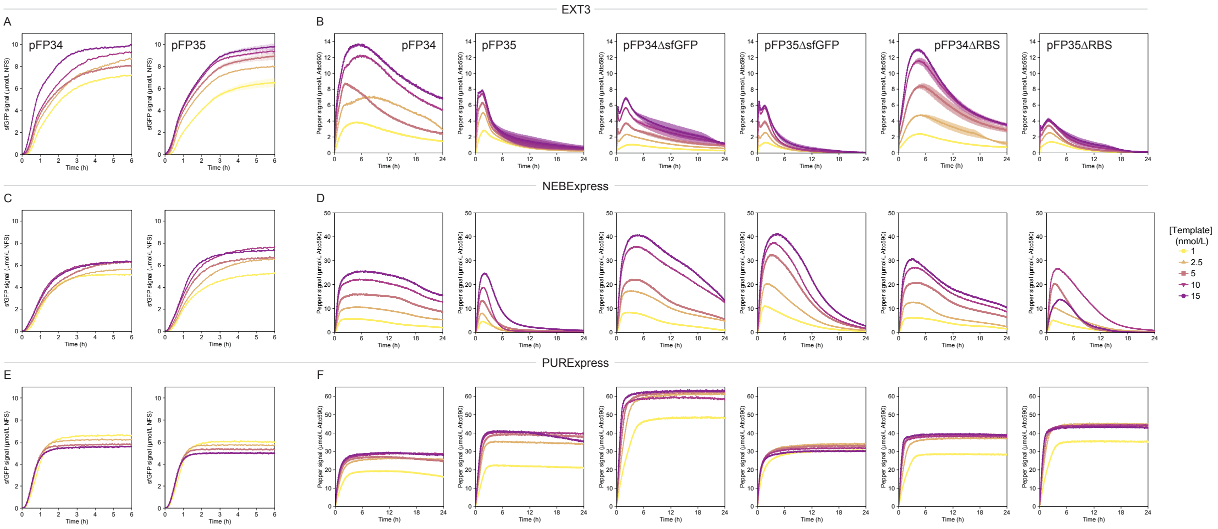

Figure S4. Measurements of translation and transcription dynamics at multiple concentrations of DNA templates in EXT3, NEBExpress, and PURExpress. **(A, C, E)** sfGFP measurements are reported in Molecules of Equivalent Soluble Fluorochrome (MESF) for a NIST-traceable fluorescein standard (NFS) and are shown for only the first 6 h of the reaction to highlight differences among reactions. **(B, D, F)** Pepper mRNA measurements are reported in MESF for Atto 590. Note the difference in y-axis ranges. For reactions in EXT3, the error bars indicate the standard deviation of three technical triplicates. No technical replicates were run for reactions in NEBExpress or PURExpress.

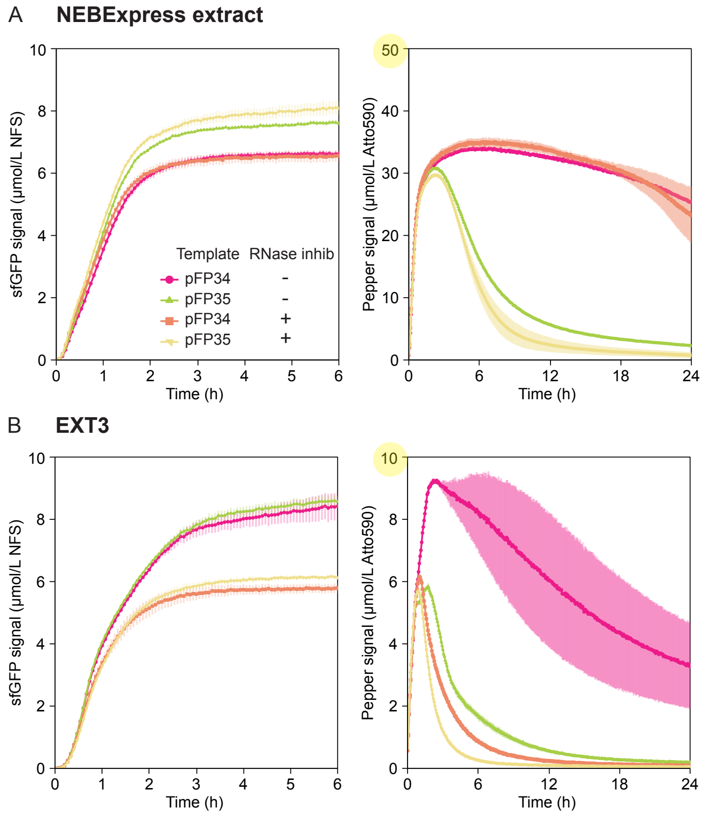

Figure S5. Measurements of transcription and translation dynamics in **(A)** NEBExpress extract and **(B)** EXT3 with and without added RNase inhibitor at 0.8 U/µL. All reactions include a DNA template at 5 nmol/L. sfGFP measurements are reported in Molecules of Equivalent Soluble Fluorochrome (MESF) for a NIST-traceable fluorescein standard (NFS) and are shown for only the first 6 h of the reaction to highlight differences among reactions. Pepper mRNA measurements are reported in MESF for Atto 590. Note the difference in y-axis ranges. The error bars indicate the standard deviation of three technical triplicates.

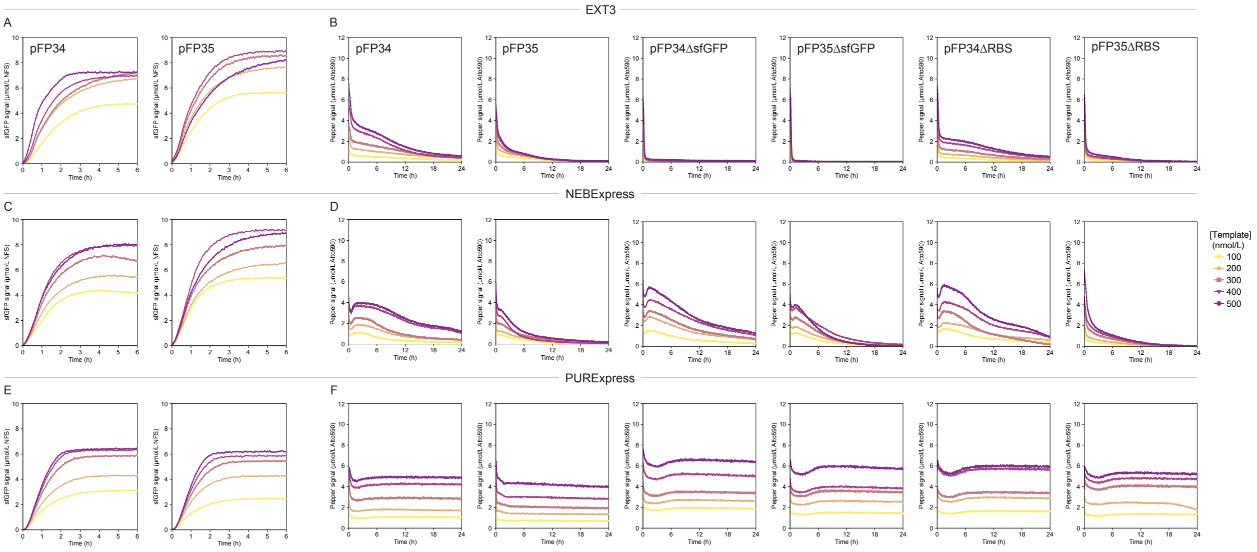

Figure S6. Measurements of **(A)** translation and **(B)** transcription dynamics at multiple concentrations of mRNA templates in EXT3, NEBExpress, and PURExpress. An mRNA concentration of 300 nmol/L was used in the experiments described in the main text because this concentration yielded a similar maximum sfGFP signal in EXT3 as DNA templates. sfGFP measurements are reported in Molecules of Equivalent Soluble Fluorochrome (MESF) of a NIST-traceable fluorescein standard (NFS) and are shown for only the first 6 h of the reaction, because the signal remains constant until 24 h. Pepper mRNA measurements are reported in MESF for Atto 590. No technical replicates were run for this experiment.

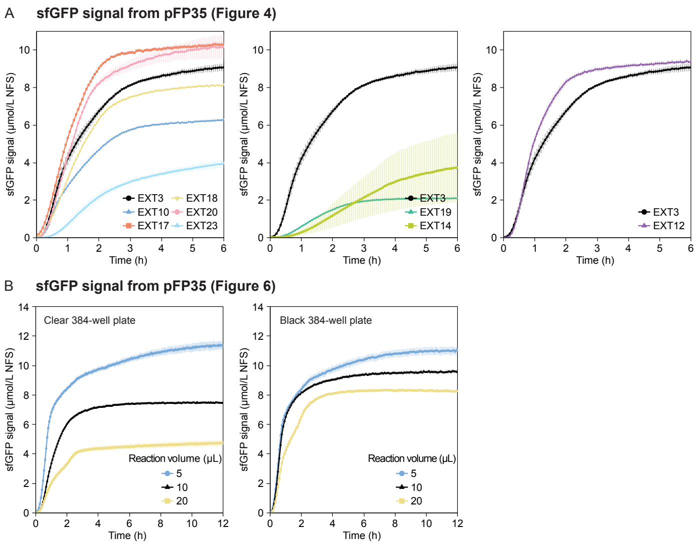

Figure S7. Measurements of translation dynamics with pFP35 as the DNA template corresponding to experiments shown in **(A)** Figure 4 and **(B)** Figure 6. These data were omitted from Figures 4 and 6 due to the similarity between the sfGFP signals generated with pFP34 and pFP35. sfGFP measurements are reported in Molecules of Equivalent Soluble Fluorochrome (MESF) of a NIST-traceable fluorescein standard (NFS). The error bars indicate the standard deviation of three technical triplicates.

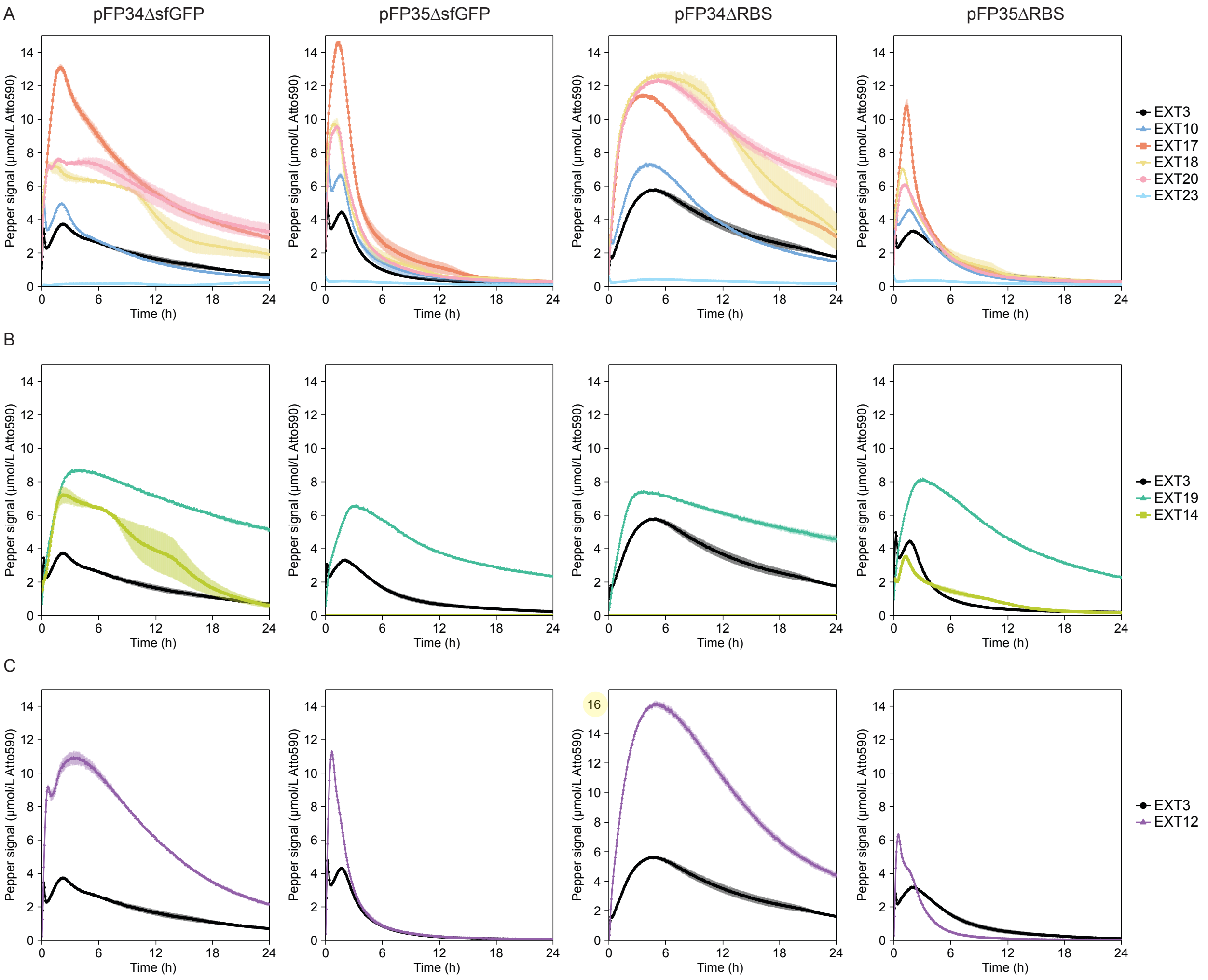

Figure S8. Measurements of transcription dynamics in extracts prepared in house **(A)** under different conditions, **(B)** under suboptimal conditions, and **(C)** from different *E. coli* host strains. Each panel includes the Pepper signal from pFP34ΔsfGFP, pFP35ΔsfGFP, pFP34ΔRBS or pFP35ΔRBS, with the relevant DNA template added at 5 nmol/L. Pepper mRNA measurements are reported in MESF for Atto 590. Note the different y-axis scale for pFP34ΔRBS in (C). The error bars indicate the standard deviation of three technical triplicates.

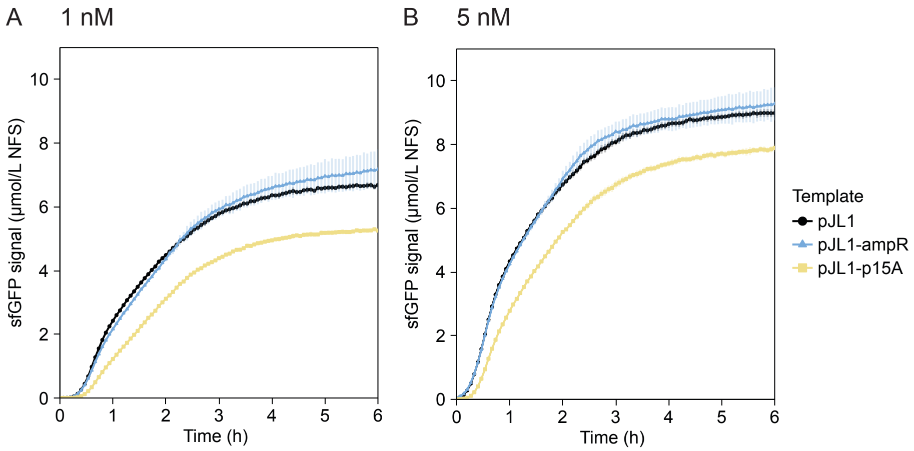

Figure S9. Measurements of translation dynamics for DNA templates with modified backbones added to EXT3 at **(A)** 1 nmol/L and **(B)** 5 nmol/L. In pJL1-ampR, the gene that confers resistance to kanamycin was replaced with one that confers resistance to ampicillin. In pJL1-p15A, the ColE1 origin of replication was replaced with the p15A origin. Changing the origin of replication reduced the rate of translation and the maximum sfGFP signal. sfGFP measurements are reported in Molecules of Equivalent Soluble Fluorochrome (MESF) for a NIST-traceable fluorescein standard (NFS) and are shown for only the first 6 h of the reaction. The error bars indicate the standard deviation of three technical triplicates.

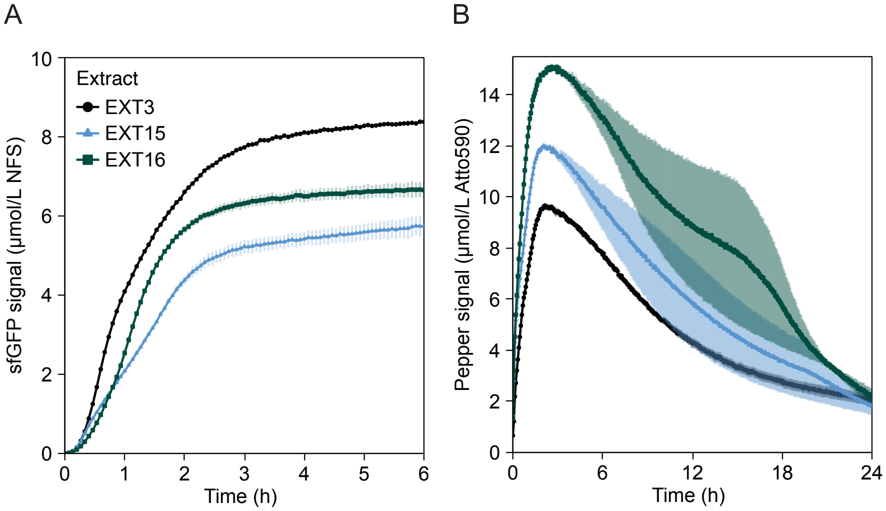

Figure S10. Measurements of **(A)** translation and **(B)** transcription dynamics in extracts derived from cells harvested in late-exponential phase (EXT15 and EXT16) with pFP34 as the DNA template. All reactions include pFP34 at 5 nmol/L. sfGFP measurements are reported in Molecules of Equivalent Soluble Fluorochrome (MESF) for a NIST-traceable fluorescein standard (NFS) and are shown for only the first 6 h of the reaction to highlight differences among extracts. Pepper mRNA measurements are reported in MESF for Atto 590. The error bars indicate the standard deviation of three technical triplicates.

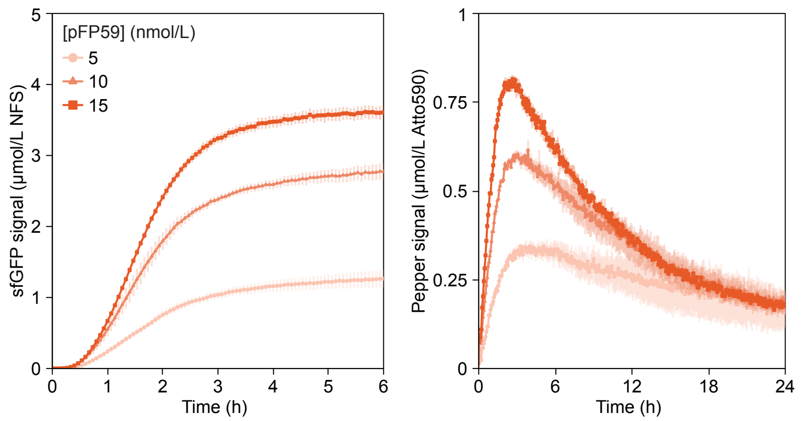

Figure S11. Measurements of translation (left) and transcription (right) dynamics in EXT3 at multiple concentrations of pFP59. sfGFP measurements are reported in Molecules of Equivalent Soluble Fluorochrome (MESF) for a NIST-traceable fluorescein standard (NFS) and are shown for only the first 6 h of the reaction to highlight differences among extracts. Pepper mRNA measurements are reported in MESF for Atto 590. The error bars indicate the standard deviation of three technical triplicates.

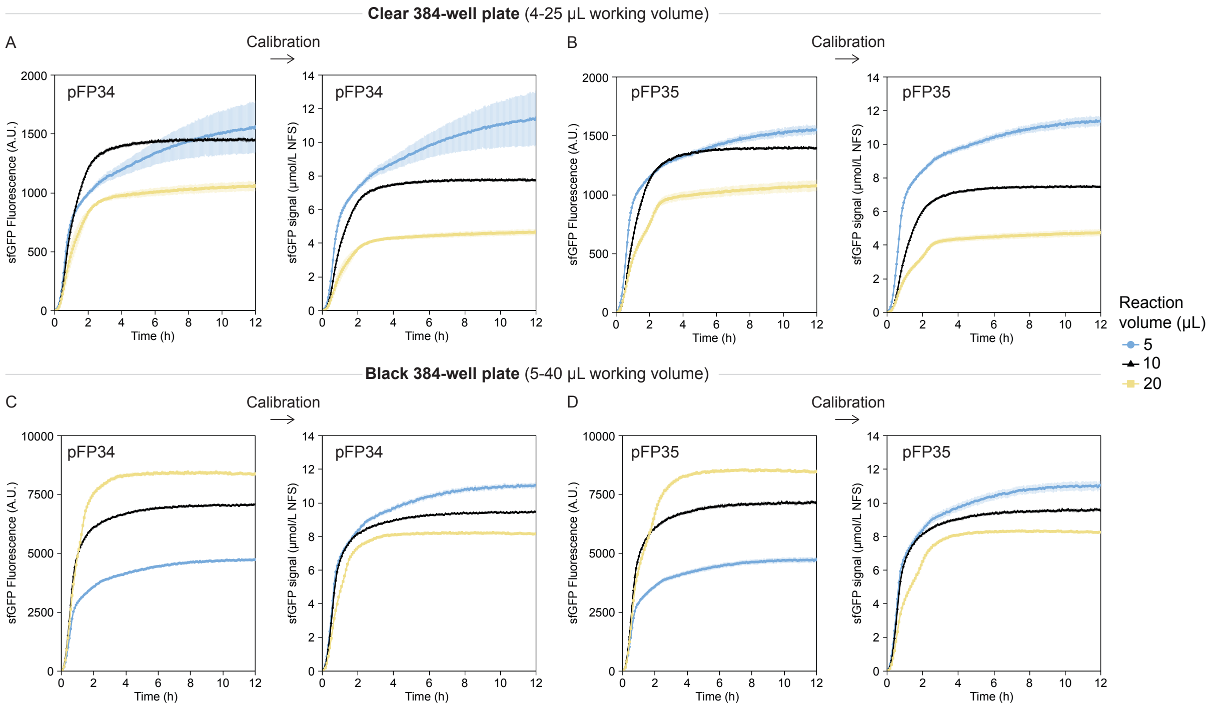

Figure S12. Measurements of sfGFP fluorescence intensity in arbitrary units (A.U.) generated in **(A,B)** a clear 384-well plate or **(C,D)** a black 384-well plate with pFP34 or pFP35 DNA templates at multiple reaction volumes in EXT3. Fluorescence intensity values were converted to units of Molecules of Equivalent Soluble Fluorochrome (MESF) for a NIST-traceable fluorescein standard (NFS), and are displayed in Figure 6. Signal calibration changed the trend for sfGFP signal with increasing reaction volumes in the black 384-well plate. In all experiments, the DNA templates were added at 5 nmol/L. The error bars indicate the standard deviation of three technical triplicates.

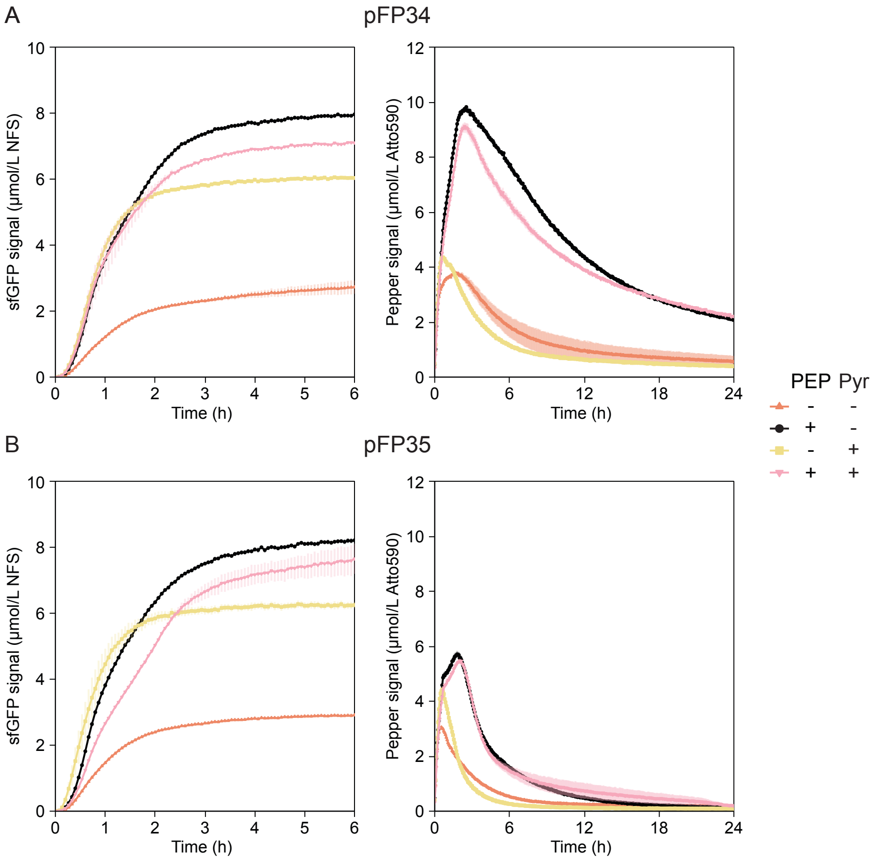

Figure S13. Measurements of transcription and translation dynamics in EXT3 supplemented with different energy substrates—phosphoenolpyruvate (PEP) or pyruvate—for ATP regeneration for expression from **(A)** pFP34 and **(B)** pFP35. In each panel, the left plot shows translation measurements, and the right plot shows transcription measurements. sfGFP measurements are reported in Molecules of Equivalent Soluble Fluorochrome (MESF) for a NIST-traceable fluorescein standard (NFS) and are shown for only the first 6 h of the reaction because the signal remained constant until 24 h. Pepper mRNA measurements are reported in MESF for Atto 590. The error bars indicate the standard deviation of three technical replicates.

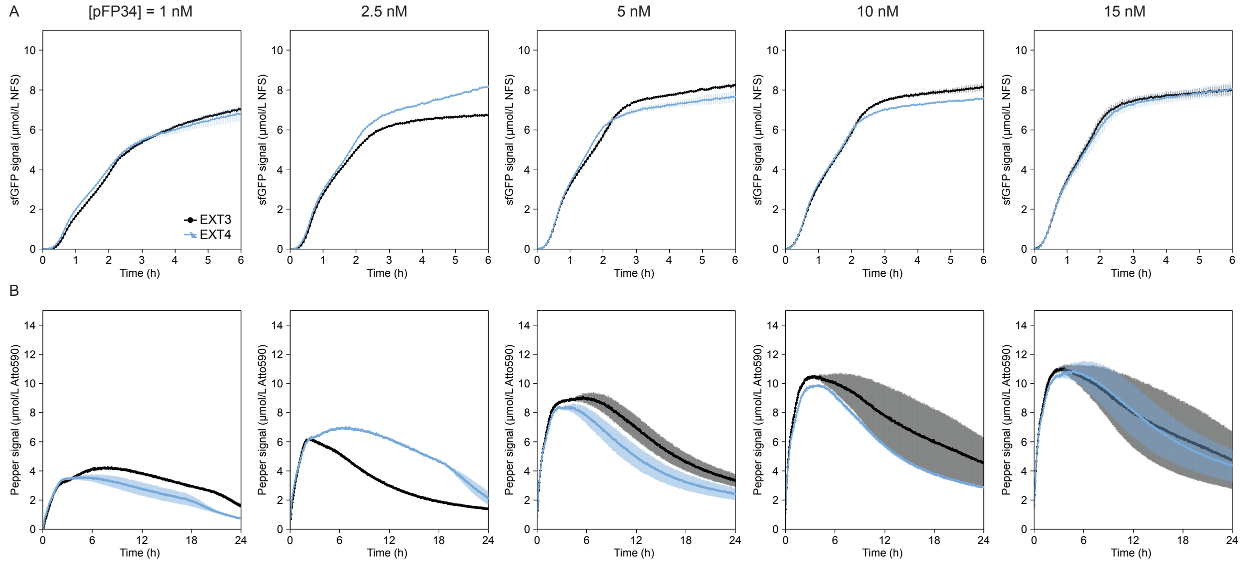

Figure S14. Measurements of **(A)** translation and **(B)** transcription dynamics from pFP34 DNA in EXT3 and EXT4, two nominally identical extracts prepared on different days. All reactions include pFP34 DNA at 5 nmol/L. sfGFP measurements are reported in Molecules of Equivalent Soluble Fluorochrome (MESF) for a NIST-traceable fluorescein standard (NFS) and are shown for only the first 6 h of the reaction. Pepper mRNA measurements are reported in MESF for Atto 590. The error bars indicate the standard deviation of three technical triplicates.

Disclaimer: Certain commercial entities, equipment, or materials may be identified in this document to describe an experimental procedure or concept adequately. Such identification is not intended to imply recommendation or endorsement by the National Institute of Standards and Technology, nor is it intended to imply that the entities, materials, or equipment are necessarily the best available for the purpose. Official contribution of the National Institute of Standards and Technology; not subject to copyright in the United States.

VI. References

(1) Zhang, Y.; Deveikis, M.; Qiu, Y.; Bjorn, L.; Martinez, Z. A.; Chou, T. F.; Freemont, P. S.; Murray, R. M. Optimizing Protein Production in the One-Pot PURE System: Insights into Reaction Composition and Expression Efficiency. *ACS Synth Biol* **2025**, *14* (5), 1496–1508. https://doi.org/10.1021/acssynbio.4c00779.
